# Ethical Considerations and Dilemmas Before, during and after Fieldwork in Less-Democratic Contexts: some Reflections from Post-Uprising Egypt

**DOI:** 10.1007/s12108-017-9363-z

**Published:** 2017-08-19

**Authors:** Arne F. Wackenhut

**Affiliations:** School of Global Studies, University of Gothenburg, Box 700, 40530 Gothenburg, SE Sweden

**Keywords:** Egypt, Fieldwork, Informed consent, Interviews, Research ethics, Social movements

## Abstract

How do we conduct ethically sound social research in less- or non-democratic settings? Here, the ‘ethical guidelines,’ or ‘codes of conduct’ outlined by our professional organizations provide some, albeit only insufficient guidance. In such contexts, issues like informed consent or the avoidance of harm to research participants have to be – based on a careful analysis of the situation on the ground – operationalized. What are, considering the particular social and political context in the field, the potential risks for interviewees and the researcher, and what can be done to eliminate or at least mitigate these risks? Reflecting on extensive fieldwork on the role of the prodemocracy movement during the Egyptian Uprising of 2011 in the wake of the so-called ‘Arab Spring,’ this study illustrates how rather abstract ethical considerations can be handled practically in an environment that is characterized by increasing levels of political repression and decreasing civil liberties. It is in such contexts that a failure to carefully consider such ethical questions entails a very real risk of endangering the livelihoods and even lives of research participants. Furthermore, it is shown that these and similar issues are not only of critical importance when designing a research project, but that they might have to be revisited and renegotiated at later stages of the research process – even after the conclusion of the data collection phase. Here, questions of data protection, anonymity of informants, and the associated ‘do no harm’ principle are particularly pertinent.

## Introduction

How can we translate the – useful but rather general – ethical guidelines of our professional organizations into practice when research is being conducted in less- or non-democratic settings? Reflecting on my fieldwork on prodemocracy activism during the Egyptian Uprising of 2011 in ‘post-Arab Spring’ Egypt, this study emphasizes the importance of operationalizing and critically reflecting on the notions of ‘informed consent’, and ‘do no harm’ before, during and after the collection of empirical data.

The American Sociological Association (ASA), the British Sociological Association (BSA) and similar organizations have drafted elaborate ‘Ethical Guidelines,’ or ‘Codes of Conduct’ to guide our work with regards to the ways in which we conduct and communicate our research. Whilst generally providing valuable advice for ethically sound conduct, they seem to provide insufficient guidance in relation to the rather particular requirements that research in ‘democratically restricted environments’ (cf. Smeltzer 2012) necessitates. Research that fails to account for the idiosyncrasies of less- or non-democratic settings entails a very real risk of jeopardizing the physical or psychological well-being as well as the livelihoods of research participants (cf. Höglund 2011).

These and similar ethical considerations are not only of utmost importance when designing research projects – to get initial approval from Institutional Review Boards (IRB) –, but also when collecting empirical data in the field. These aspects have received considerable scholarly attention within our profession, and helped stimulate a lively debate on the topic (cf. Shahidian 2001; Romano 2006; Wood 2006). Based on experiences gained in the context of extensive research on prodemocracy activism during the Egyptian Uprising of 2011, this article stresses the importance of sufficiently operationalizing the rather general guidelines, and illustrates challenges associated with fieldwork in less- or non-democratic settings.

Furthermore, while others have hinted at the importance of revisiting ethical issues (Bronéus 2011; Miller and Bell 2012), it is shown that decisions made earlier during the research process might have to be renegotiated and possibly adjusted if and when the conditions on the ground change.

Even though this article does not present solutions to these challenges that are universally generalizable across all contexts, it provides a valuable contribution to the recent debates on research ethics. Firstly, it might help to sensitize scholars for the particular requirements and challenges of designing and conducting ethically sound social research in difficult, dangerous or highly contingent settings where the stakes for participants and researchers alike are markedly higher. Secondly, it highlights a set of ethical dilemmas at the intersection of themes like informed consent, participant anonymity, ‘giving voice’ to informants and the ‘do no harm’ principle that might emerge at different stages of the research process. Here, the present article might help researchers confronted with similar situations to make a more informed decision.

For this purpose, the article is structured as follows. A first section provides a brief overview of my research project on the role of prodemocracy activists during the Egyptian Uprising of 2011. Subsequently, I sketch ou the social and political situation in the country during the fieldwork, which represented a rather particular set of challenges for the ethically sound conduct of social research. Following this discussion, I outline – in broad strokes – some of the key ethical guidelines of our profession, namely the avoidance of harm to potential research participants and informed consent, as far as they have been discussed in the literature more generally and more specifically in relation to ‘difficult contexts.’

Then, I reflect on the ways in which I sought to uphold these rather broad guidelines in the context of my own work on Egyptian prodemocracy activism. This includes not only the process of gaining the truly informed consent of prospective interviewees, the protection of the integrity of collected data, but also the avoidance of harm to research participants as well as myself. Here, I also touch upon the issue of how I presented and conducted myself with regards to my research participants on the one hand and outsiders on the other. Subsequently, having discussed some of the key ethical challenges during the data collection phase, I touch upon a number of challenges that emerged after the conclusion of fieldwork. These are, namely the dilemma of wanting to give voice to my informants in the material as opposed to a need to anonymize their identities given a changed political situation in the country. A final section then summarizes the main findings and elaborates on the more general implications for our profession if data collection occurs in difficult contexts.

## Researching Prodemocracy Activism in Post-Uprising Egypt

My research has to be understood in the context of the popular uprisings that spread across the Middle East and North Africa region in the wake of Mohamed Bouazizi’s self-immolation in Tunisia on December 18, 2010. At that point few scholars expected that this act of despair, which followed a public humiliation by local officials, would represent the spark setting off a transnational protest wave, which resulted in the toppling of long-time authoritarian rulers in countries like Tunisia, Egypt, and Libya.^1^ In the Egyptian case, protesters succeeded in ousting President Hosni Mubarak in a matter of eighteen days (cf. Lynch 2012). This protest episode paved the way for the first free and democratic elections in the country’s modern history. Considering that elements within the Egyptian prodemocracy movement (El-Mahdi 2009) had unsuccessfully tried to mobilize for significant socio-political change in the country for the better part of a decade (cf. Abdelrahman 2015), I embarked on a project trying to understand the mobilization process leading up to and the diffusion of collective contentious behavior during the so-called January 25 Uprising. Building upon and seeking to contribute to the broader contentious politics framework (cf. McAdam et al. 2001; Tilly and Tarrow 2007), I sought to better understand the diffusion of protest during this uprising, which came to involve a broad cross-section of the Egyptian society. At the same time, I hoped to improve upon the conceptualization of causal mechanisms in the contentious politics framework, which was criticized rather heavily by, for example, Ruud Koopmans (2003) on the grounds of insufficient specification and theorization.

To realize these research objectives, I designed an in-depth single case study (cf. Yin 2009) using ‘explaining-outcome process tracing’ (Beach and Pedersen 2013) and semi-structured interviews (cf. Bronéus 2011) with members of what Kilian Clarke (2014) called the Cairo-based political opposition – a network of different social movement organizations and nongovernmental organizations (NGO) engaging in human rights advocacy.

Fieldwork was mostly conducted in and around Cairo between late 2014 and 2015. This means that it occurred not only after the fall of Hosni Mubarak who had been in power for nearly thirty years, but also after the relatively short-lived presidency of democratically elected Muslim Brotherhood candidate Mohamed Morsi. Thus, the bulk of the empirical data was collected in a period of narrowing political opportunities, and in the midst of a crackdown by the military-backed regime of President Abdel Fattah el-Sisi on civil society in general, and on prodemocracy activists in particular.

## Political Opportunities and Threats in Post-Uprising Egypt

Ever since the June 30 protests in 2013 and the following *soft coup*, which resulted in the ouster of President Mohamed Morsi and the Muslim Brotherhood’s Freedom and Justice Party, the conditions for activists within the Cairo-based political opposition deteriorated markedly. In the terminology of Goldstone and Tilly (2001), one might argue that the situation in the country was increasingly characterized by diminishing opportunities for collective contentious claim-making, whilst both the current and repressive threat increased substantially.^2^

These events marked the beginning of a wave of political repression that initially resulted in the so-called *Rabaa al Adawiya Square massacre* on August 14, 2013. It left hundreds of people, mostly supporters of Morsi, but also members of the security forces, dead.^3^ In the following months, thousands of activists were imprisoned and hundreds of Muslim Brotherhood members were handed death sentences.^1^

However, soon thereafter, this crackdown was no longer limited to supporters of the Islamist Muslim Brotherhood, but increasingly targeted other segments of civil society as well. New legislation, like the so-called *protest law*, has been used to prosecute and imprison civil society and prodemocracy activists (Daily News Egypt 2014). While laws governing the freedom of assembly are a common occurrence in many – if not most – polities, the text of this Egyptian law –and its practical implementation– are often regarded as rather harsh and drastic (Human Rights Watch 2013). For instance, the law functioned as the legal basis for the arrest of prominent activists like Ahmed Maher (Mada Masr 2013) who was sentenced to a three-year prison sentence for “protesting without permission and assaulting police” (BBC 2017).^4^ As, for instance, Nadeen Shaker (2016) notes, hundreds of activists suffered a similar fate. Shaker (2016) mentioned an “estimated 16,000 political prisoners behind bars in Egypt [,while l]eading human rights organizations believe the number is considerably higher.”

Some NGOs, like Human Rights Watch, estimate that there are currently about 40,000 political prisoners (cf. Stork 2015). This number is disputed by government officials, and data collected by the Egyptian Commission of Economic and Social Rights (ECESR), which was discussed by Shaker (2016), suggests that “some 41,000 Egyptians have been arrested or faced criminal charges for political reasons” ever since Abdel Fattah el-Sisi became president. Moreover, since the second half of 2015, local human rights NGOs have been reporting an increasing number of *forced disappearances* (Daily News Egypt 2016). According to the Rome Statute of the International Criminal Court (ICC), the practice of forced disappearances refers to

arrest, detention or abduction of persons by, or with the authorization, support or acquiescence of, a state or a political organization, followed by a refusal to acknowledge that deprivation of freedom or to give information on the fate or whereabouts of those persons, with the intention of removing them from the protection of the law for a prolonged period of time (UN General Assembly 1998).

A report compiled by Amnesty International (2016) mentions hundreds and includes detailed accounts of 17 cases of such forced disappearances.

Therefore, bearing in mind the high repressive threat and the rather complex political situation on the ground, it was necessary to pay close attention to ethical and security considerations for the purpose of eliminating or at least minimizing the very real risk of *harm* to research participants. At the same time, matters of personal security had to be taken into account as well. Not only had an established scholar working on Egyptian politics been barred from entering the country in late 2014 (Kingsley 2014), but others, like a French M.A. student conducting research on youth activism in the country, had been detained and subsequently deported (AFTE 2016). Before elaborating on the ways in which these challenges were addressed in the context of my own research project, the following sections spell out a number of overarching ethical considerations for social research in general, and research in difficult or ‘democratically restricted’ (cf. Smeltzer 2012) contexts in particular.

## On the Ethically Sound Conduct of Social Research in Difficult Contexts

Every scholar whose research involves humans as research subjects will have to contemplate and carefully consider the ethical implications of a planned project. In most, but not all, contexts the approval of an Institutional Review Board^5^ marks a necessary albeit insufficient hurdle in the process of designing sound ethical foundations for such an endeavor.

Arguing that the research ethics is an underresearched area would be a grave mischaracterization of the current state of affairs. In fact, in the period between 1987 and 2016, *The American Sociologist* has featured no less than 232 articles that have touched upon issues of research and professional ethics in one form or the other.^6^ Here, issues relating to the ethics and ethical dilemmas of field research in difficult settings deserve particular attention. Some of them are common to all forms of social research, but a subset is more important when research is conducted in difficult, potentially dangerous, and often less-democratic contexts. In my own research in the Egyptian context, three broader themes emerged as particularly important and problematic. These three are, firstly, the ‘*do no harm*’ principle, secondly, questions of *informed consent*, and lastly, issues regarding the *anonymity of sources*. In the following, each of these aspects will be discussed and put into the context of previous scholarship more generally, and, in particular, that tackling these questions in relation to fieldwork in difficult settings.

## The ‘do No Harm’ Principle and Security Considerations in the Field

At the very foundation of our work as sociologists lays what Bryman (2008) called the ‘do no harm’ principle. In the ethical guidelines of the American Sociological Association ( 1999), this principle can be found under item 2 (d), which urges sociologists to refrain from

undertaking an activity when their personal circumstances may interfere with their professional work or lead to *harm* for a student, supervisee, *human subject*, client, colleague, or other person to whom they have a scientific, teaching, consulting, or other professional obligation (ASA 1999, italics my own).

At first sight, these guidelines appear to provide clear guidance for researchers. However, Bryman (2012) rightfully asks what exactly harm is. Drawing on the work of Diener and Crandall (1978), he notes that it might ‘entail a number of facets: physical harm; harm to participants’ development; loss of self-esteem; stress’ and other aspects.

Providing some ‘Reflections on Dangerous Fieldwork’ Patrik Peritore (1990), drawing on his work in the Latin American context, addresses the avoidance of harm to research participants in great detail. In this discussion, he devotes significant attention to questions concerning the security of research participants. Using the example of his fieldwork in Brazil, he warns that certain precautions and constant vigilance are of paramount importance, since

From 1964 to 1985, Brazil’s network of 16 secret police forces assisted by 250,000 paid informants, an apparatus set up with CIA assistance, systematically persecuted “subversives” and “leftists”. Thousands of persons were killed, or “disappeared” to underground prisons and torture centers (Peritore 1990).

Operating in this context, Peritore (1990) outlines how he, due to the type and nature of his research, became a target for the local security services, and came, at times, under personal and digital surveillance. Similarly, in his discussion of field research in the Middle East, Romano (2006) warns that respondents, in certain contexts, may ‘risk arrest, torture, or execution for speaking with you.’ Romano (2006) implores researchers to take steps to ensure the protection of identities and information provided by research participants. Discussing similar risks in the context of fieldwork and in-depth interviews in conflict or post-conflict situations, Bronéus (2011) argues that it is

important to make an ethically informed risk assessment concerning how to access interviewees in the safest possible way, and how to ensure the participants’ security and confidentiality during and after the interview (Bronéus 2011).

Considering that it might be, given the political situation on the ground, impossible to completely eliminate such risks to research participants, it is all the more important that prospective interviewees can make an informed decision whether or not they want to volunteer their time and expertise.

## Informed Consent

Therefore, with few exceptions, *informed consent* ought to represent another cornerstone for the conduct of social research. Discussing informed consent, Bryman (2012) advocates that ‘prospective research participants should be given as much information as might be needed to make an informed decision whether or not they wish to participate in a study.’ At first sight, this requirement appears rather unambiguous, but several scholars have problematized a number of aspects relating to questions of when informed consent actually represents *informed* consent. For instance, feminist scholars warned about the impact of hidden power relations between researcher and subject, and how they might unwittingly coerce participation (Ribbens and Edwards 1998; Miller and Bell 2012). Others have urged for a cautious approach when dealing with ‘special populations’ including, for example, the ‘mentally impaired’ whose capacity to fully grasp the purpose of a study and their role in it might be somewhat limited (Warren and Karner 1990).

To ensure and document informed consent, some studies draw upon so-called informed consent forms, which provide participants with the information required to make a decision (Sin 2005). However, as Miller and Boulton (2007) have cautioned, individuals ‘who identify themselves as socially excluded or as belonging to a marginalized group, are unlikely to formally consent in writing to participation in a study’. Furthermore, if field research is conducted in less-democratic or non-democratic settings, as those mentioned above, informed consent forms containing personal information might incriminate and jeopardize potential research participants – thus violating the ‘do no harm’ principle. Thus, gaining true and un-coerced informed consent of participants in potentially difficult settings represents a non-trivial challenge that requires substantial pre-planning and deep knowledge of the local conditions in the field.

This particular point also touches upon the issue of respondent anonymity. The ASA (1999) ‘Code of Ethics’ puts a high premium on this particular point, by arguing that

Sociologists *do not disclose* in their writings, lectures, or other public media confidential, *personally identifiable information concerning their research participants* (…) or other recipients of their services which is obtained during the course of their work, *unless consent from individuals or their legal representatives has been obtained* (ASA 1999, *italics* mine).

Thus, it is of critical importance to implement measures to ensure the anonymity of respondents after, but also during the data collection stage. However, at the same time, should informants desire to be identified in the material, researchers should try to, if possible, respect these wishes. Peritore (1990) and others have alluded to the potentially extractive nature of fieldwork, and urged scholars to try to reciprocate the informants’ efforts. Even though scholarly articles seldom attract the readership of more journalistic accounts, respecting the wishes of informants to be identified in the material could be regarded as one possible way to reciprocate simply by giving voice to otherwise silenced groups or populations.

Closely related to these types of questions are issues of deception in the context of social research. Not least since Milgram’s (1965) experiment on obedience to authority, deception represents a controversial and usually condemned strategy in our field (cf. Leo 1996, 1995; Erikson 1995). The ASA (1999) ‘Code of Ethics’ strongly advises against the use of deception in research, with few notable exceptions that are highly context dependent. In short, unless the deception is *harmless*, represents the only practicable way of conducting a given project, and is furthermore unlikely to affect the participants’ willingness to participate ‘[s]ociologists do not use deceptive techniques’. Conducting research in the broader Middle East, Romano (2006) apparently opted for a mixed approach as far as deceptive techniques are concerned. Towards his informants and research participants he was open and honest about his role as a researcher, and the aims and purpose of his research project. This might not necessarily have been the case when dealing with authorities in the countries where he conducted his research, occasionally creating the impression of a ‘dim-witted […] young Canadian tourist’ for the purpose of, for instance, resolving a potentially problematic situation with members of the Iranian Revolutionary Guard. While certainly not the most honest or desirable approach to research ethics, a certain and selective degree of deception towards individuals and authorities not directly involved in the research process might very well be the only feasible and reasonably *safe* way of conducting social research in less-democratic or similarly difficult contexts.

## Ethical Considerations before and during Field Research in Post-Uprising Egypt

Having carefully studied the recommendations of the ASA, BSA and similar bodies, *informed consent* was made to be one of the cornerstones guiding the data collection phase of my inquiry into the role of the prodemocracy movement during the Egyptian Uprising of 2011. Considering, however, the ways in which the political situation in Egypt had developed in the aftermath of the June 30, 2013 *coupvolution*, I decided that a written consent form – as suggested by Bryman (2008) – would be infeasible in the context of the rising repressive threat for many members of civil society and prodemocracy social movement organizations. To minimize the likelihood of unintentionally harming my interviewees by requiring them to fill in such forms, I decided to ensure their informed consent verbally without leaving a potentially compromising paper trail. A two-pronged approach was designed and implemented to make sure that every potential interviewee could make an informed decision whether or not to participate in this project.

Here, the recruitment process represented the first step. When reaching out to prospective interviewees I made sure, following the recommendations of Bryman (2012), to explain 1) my own professional background and institutional affiliation, 2) the aims and purpose of the research project, that 3) I would be using the interview material for the purpose of my research project, 4) that their participation would be strictly voluntary, and 5) that I would take all feasible measures to ensure their anonymity in my material, should they wish to remain anonymous.

In cases where prospective participants indicated that they were not interested in being interviewed for the project, I thanked them for their time and refrained from contacting them again to respect their privacy. However, when a prospective participant agreed to meet for an interview, I proceeded by scheduling an appointment that best suited their own needs and personal preferences in terms of time and meeting place. Interviews were conducted in various localities, like their homes, offices of human rights NGOs, or smaller cafés in various parts of Cairo.^7^ At the beginning of each interview I made sure to revisit the aforementioned five points to ensure that the informants were able to make an informed decision whether or not they wanted to proceed with the interview. When informants declined the offer to remain anonymous in the material, I asked, in a follow-up question, if they would agree to be quoted by name in the material. This served the purpose of making their consent on this particular point not only informed, but also explicit. Additionally, I inquired if they had any objections to me electronically recording^8^ our conversation for the purpose of later transcribing and coding it based on a number of theory-guided themes and codes.

Out of a total of 69 semi-structured interviews that were conducted in the process of this research project, only three interviewees asked to remain anonymous in the material, and two informants objected to an electronic recording of the interview. While a majority of interviewees had no clear preference either for or against anonymity, a number of respondents actively asked to have their real names in the material and in subsequent publications. This stemmed from a wish to make their own voices heard on the subject matter of the research project. In the two cases where informants asked to not have the interview recorded, extensive notes were taken during the interview and subsequently transcribed digitally in close temporal proximity to the interview situation.

To *protect the integrity of data* gathered in interviews, and to ensure that respondents were neither identified nor identifiable prior to, during and after the research visits to Cairo, ‘Operations Security’ (OPSEC) was considered a high priority throughout the process. Interview material, like the aforementioned voice recordings, were stored on an encrypted and password protected hard drive,^9^ and contained no information that would have allowed for the identification of research participants. In this material as well as the digital transcripts five-digit respondent identifiers were used to conceal the identities of the respondents. Contact information was kept on a separately stored and encrypted as well as password protected flash drive.

While the protection of interviewees was, as shown above, a primary concern throughout the fieldwork in Egypt, matters of personal security were deemed important as well. Building on the experiences of scholars like Peritore (1990), or Romano (2006), a number of precautions were taken to reduce risks to my own safety throughout the data collection phase.

This is, however, not to say that Egypt should be considered – at least at the time when the majority of the fieldwork was conducted – a particularly dangerous or high-risk place for international visitors. Already in 2014, the regime was confronted with a growing insurgency by a group called *Ansar Bait al-Maqdis*, which declared its allegiance to the so-called Islamic State in November of 2014. Already then there were reports of an increasing number of attacks on security personnel. However, at the time these attacks were predominantly limited to the northern parts of the Sinai Peninsula. Overall, crime rates were rather low, and travel recommendations by, for example, the Swedish and German ministries of foreign affairs mostly contained advice typical for international travel in the post-9/11 world. They included a call for vigilance near public buildings, and cautioned travelers to avoid, if possible, large public gatherings or congregations of people (Federal Foreign Office 2017).

This seems like sound advice for the general traveling public, but considering that I was going to interview individuals that were the more or less explicit target of a regime crackdown on oppositional and civil society actors, I decided to take some additional precautions in this context.

These measures could be conceived of along the lines of more general preparations and precautions, as well as daily routines designed to minimize personal risks. In terms of the more general precautions, I made sure to contact the embassy and to notify them of my presence in the country and the purpose of my research. Additionally, I saved relevant telephone numbers for any sort of emergency on my mobile phone.

With regards to the more daily routines it seems worthwhile to address the issue of deception once more, which was discussed previously. As noted earlier, the informed consent of my research participants represented an important part of this study. Thus, potential and actual interviewees and respondents were openly and honestly informed about the purpose of my study, and how I would use information even before they volunteered to participate in my research. However, considering the extent and influence of the *Mukhabarat* (secret police) with its extensive network of informants (cf. Marfleet 2009), I chose a more selective approach to the truth as far as individuals who were not research participants were concerned. Up to a certain extent, I became the embodiment of the ‘dim-witted […] young’ (cf. Romano 2006) German tourist, who was in the country to get to know the country and its citizens. While, generally speaking, deceptive techniques should be regarded as highly problematic and undesirable, this selective deception seemed to be the only viable option in this particular setting and context. Overall, given the political climate combined and a growing media discourse seeming to instigate a fear of foreign (i.e. U.S.) interference in the country, I – a tall, blond and blue-eyed white male – was occasionally met with suspicion as I was going about my daily business. More often than not, taxi drivers would inquire about my nationality with a somewhat distrustful ‘*American?’* By asking me whether or not I was an American citizen, they seemed to not only express their loathing for the United States of America, but also to suggest that I might be a foreign spy intent on harming the country. Usually, by truthfully responding that I was a German citizen, the conversation quickly shifted towards the ingenuity of German car manufacturers, or the latest exploits of German soccer teams in the UEFA Champions League.

Similarly, one particular street hawker, who had his usual place of business close to my lodgings, became increasingly interested in my person and my activities. Knowing full well that some street hawkers work as police informants, I made sure to tread carefully around him. A few weeks into my first stay in Cairo, our interactions developed almost into a ritual. Nearly every time I happened to meet him, he would – after exchanging the usual pleasantries and peddling his assortment of souvenirs – ask me where I was going and what the purpose of my stay was. Then, I would usually tell him that I was in the city to get to know the country, its inhabitants and to improve on my Arabic. One time, when he directly and half-jokingly (?) asked me if I was a ‘spy,’ I countered by jokingly proclaiming that I was a high value asset of a foreign intelligence agency and hatching some evil plot.^10^ This riposte immediately defused the situation and we resumed our ritualized conduct. In retrospect, and especially when considering the gruesome fate of Giulio Regeni – an Italian doctoral student – and his premature and unnecessary death, this approach of selective deception seemed like the only viable option given the situation on the ground.^11^

## Ethical Challenges after Fieldwork

While it would be a misrepresentation to characterize the political situation in the country, especially for actors in the prodemocracy movement and human rights NGO sector, prior to and during the field work in 2014 and 2015 as open and unproblematic, it could be argued that the situation deteriorated markedly after the conclusion of the data collection phase in December 2015. This deteriorating situation, which could at least partly be interpreted as a successful repression of this type of activism (cf. Tarrow 2011), found its expression in a number of different ways. For instance, in early 2016 the Italian doctoral student Giulio Regeni, who was writing his Ph.D. thesis on the role of the unofficial labor movement and unionization attempts in post-revolutionary Egypt, was abducted, tortured and killed shortly after the fifth anniversary of the January 25 Uprising. While, at the time of writing, the official investigation is still ongoing, a number of leads point towards the role of the Egyptian security apparatus in Regeni’s death (BBC 2016).

While this arguably extreme example illustrates the potential risks for foreign nationals conducting social research on a sensitive topic in the Egyptian context, local activists are confronted with a whole spectrum of repressive measures. This narrowing political opportunity structure has compelled some activists to either flee the country or to refocus their efforts on less contentious areas, like environmental conservation (Interview #1). Others have, seemingly, suspended or completely given up on their activism. A number of activists that were interviewed in the context of this project have, in the meantime, deleted their social media profiles, which used to be an important avenue for them to voice their contentious claims and disapproval of the ruling regime.

Additionally, several activists and NGO workers that were interviewed in the context of this project were arrested and at least temporarily incarcerated suspected of, for example, having violated the aforementioned *protest law*, spreading false news, or plotting to overthrow the government and alter the Egyptian Constitution. The majority of these cases occurred in the wake of popular protests in early 2016 against the planned transfer of sovereignty of two Egyptian Islands (Tiran and Sanafir) to Saudi Arabia (Michaelson 2017).

This deteriorating situation on the ground after the end of the data collection phase presented yet another set of ethical dilemmas and challenges that had to be navigated in the process of writing up the central findings. As noted earlier, the avoidance of harm to research participants, informed consent and anonymity of informants – if requested – were the central ethical guideposts before and during the fieldwork. With few exceptions, my research participants had, making an informed decision, declined the offered anonymity. In fact, as noted earlier, a number of them explicitly asked to be represented with their full names in my material.

However, given the deteriorating situation in the country, respecting the interviewees’ wishes might potentially jeopardize their material, psychological and physical well-being. This would mean that by honoring a commitment made during the fieldwork at time t_0_ I could potentially violate the ‘do no harm’ principle due to the changed situation at time t_1_. At the same time, by simply anonymizing the identities of all informants I would deprive them of their voice in the collected material, and potentially violate the terms under which they consented to be interviewed for the purpose of my project. This represented a non-trivial dilemma that was rather difficult to resolve.

Obviously, it would have been the desirable solution to simply contact the interviewees and to ask them for their opinion on the matter, since they could be expected to be better able to assess the situation on the ground and the risks associated with being named in scholarly publications. Already in the planning stage of the project there were plans for an additional research visit to Cairo to discuss my material and an almost completed manuscript with several informants. This visit would have been the ideal opportunity to discuss and probably jointly decide on a way to handle the changed situation and emerging questions of respondent anonymity. Originally, this concluding visit was not only intended as another means of triangulating my data and central findings (cf. Miles and Huberman 1994), but also meant as a way to give something back in return for the time and effort that respondents devoted to my project. This form of feedback was supposed to, at least begin to, counteract the somewhat extractive nature of fieldwork, in which researchers collect their data without necessarily reciprocating sufficiently (Peritore 1990). Unfortunately, this concluding research visit had to be canceled in the wake of the aforementioned Giulio Regeni incident and changed (i.e. more restrictive) university policies on field research in Egypt. Thus, this particular avenue of resolving the present ethical dilemma was closed for all intents and purposes.

Even contacting interviewees by email or via social media networks like Facebook or Twitter seemed infeasible without potentially jeopardizing their safety, since the domestic intelligence services have, in the meantime, significantly expanded their surveillance capabilities in the cyber domain (Ezzat 2014).^12^ Thus, with no viable means of contacting all informants in question, anonymizing the identities of all research participants seemed like the only feasible option in this context. The avoidance of harm was regarded comparatively more important than using the full name as some interviewees had requested. While there is a chance that this decision might be regarded as patronizing, it seemed to be preferable to the alternative of putting one or more participants in harm’s way.

Rather than using the five-digit respondent identifiers, the actual process of anonymizing the interviews then proceeded with the help of a list of the hundred most common first names in Egypt, which were randomly assigned to the interviewees.

## Conclusion

In conclusion, it could be argued that the established ethical guidelines of our professional associations provide useful but not necessarily sufficient guidance to ensure that research in less- or non-democratic settings upholds crucial ethical standards. Just like we have to operationalize our theoretical concepts – transforming them into variables that can be measured and observed in the empirical world – it is necessary to carefully reflect on the broader ethical implications of our research endeavors. Simply being aware of ethical guideposts like ‘informed consent,’ or ‘do no harm’ is not enough. This holds true for social research generally, but it is of critical importance when our work is conducted on sensitive topics, or in ‘democratically restricted environments’ (cf. Smeltzer 2012) where the stakes are – generally speaking – much higher. Thus, it is of paramount importance to operationalize such general guidelines and recommendations for the specific environments. This process requires careful attention to the social and political context within which potential research participants operate. It is this context that ultimately dictates the protocols and procedures we use to ensure – and record – informants’ informed consent, as well as to collect, store and present data in a way that eliminates or at least minimizes risks to participants and ourselves. At the same time, we should try to be mindful of the potentially extractive nature of fieldwork and do our best to reciprocate.

The ethical challenges that emerged throughout this research project on prodemocracy activism during the Egyptian Uprising of 2011 illustrated these aspects quite forcefully. However, the particular choices made and procedures adopted for this project to uphold ethical standards might not necessarily be directly applicable or generalizable to other settings and contexts. Here, it is the obligation of every responsible researcher to carefully consider and critically engage with these issues before, during and even after fieldwork.

## Footnotes

1 As noted by, for example, Charles Tripp (2013), the ‘Arab Spring’ as a regional phenomenon came to encompass some degree of popular mobilization in about 19 different countries. Out of these 19, large-scale protest episodes erupted in countries like Tunisia, Libya, Yemen, Bahrain, or Syria. In some of these countries, like Bahrain, protests were successfully suppressed by the regime, while other cases, Syria comes to mind, have developed into protracted armed conflicts.

2 In the political opportunity structure perspective, the notion of *current threat* denotes the “harms that are currently experience”, while the *repressive threat* captures the costs or burdens that the initiation of protest would entail. It is the combination of these two factors and perceptions of *opportunity* that that largely determine forms and extent of collective contentious behavior (cf. Goldstone and Tilly 2001).

3 The numbers of casualties vary widely. While the Egyptian Ministry of Health put forward a total of 638 deaths during the dispersal (cf. Daily News Egypt 2013), the NGO Human Rights Watch refers to 817 deaths during this incident (Human Rights Watch 2014).

4 Ahmed Maher was released from prison on January 5, 2017 after serving out his prison term, but remains –at the time of writing– under judicial supervision (cf. BBC 2017).

5 While some degree of ethical oversight over social research is useful if not necessary, a number of scholars are increasingly critical of the institutional structures that have formed in many contexts to facilitate this function. The issue of institutional review boards has been hotly debated in the scholarly community. See, for example, the work of Babb et al. (2016), or Hessler et al. (2011) who make a case for limiting the reach of these bodies.

6 The number of 232 articles was identified with a simple text search using the search term ‘research ethics’ in the online archive of the journal. This, arguably, imperfect measure, and the total count captures not only articles tackling issues related to ethical concerns in relation to field research, but also other aspects of our profession. It is, nevertheless, a clear indication of the fact that research ethics are and should remain a key concern in the ways we conduct ourselves as sociologists.

7 However, I tried to meet my interviewees in less public settings after an incident in a restaurant in Downtown Cairo where one of my interviewees wanted to meet me. During the interview, a plain-clothes police officer entered the restaurant, took several photos of us and subsequently left the establishment.

8 Since a number of interviews were conducted in public or semi-public places where a professional recording device might have raised suspicions, I opted to record interviews –given the consent of interviewees– using a voice recording application on a smartphone. Even though the sound quality of these recordings, especially in terms of background noises, compares rather unfavorably to purpose-built recording devices this seemed to be the only viable option given the political situation in the country during the field research episodes.

9 In retrospect, it might have been even more secure to refrain from physically storing the data on physical devices whilst in the field, and to directly transmit this information to a secure server using a virtual private network (VPN) connection. This might have helped to eliminate, or at least minimize, the risk of unauthorized access to the encrypted data in case the hard drive had been seized by authorities.

10 I do not fully recall whether this plot involved the wholesale theft of the Pyramids of Giza or alternatively the Sphinx.

11 At the same time, I have to admit that I felt and continue to feel deeply uncomfortable about being dishonest and deceitful towards others. Since early childhood, my parents have always put a strong emphasis on honesty and integrity.

12 Furthermore, since I only possessed detailed contact information through these channels for a subset of my interviewees, it would have been impossible to get reassurance from all of my research participants.
